# Visual Impairment at Age 85 Predicts Subsequent Cognitive Decline at Age 90

**DOI:** 10.1093/geroni/igaa057.699

**Published:** 2020-12-16

**Authors:** Jeremy Jacobs, Jochanan Stessman

**Affiliations:** 1 Hadassah Hebrew University Hospital, Jerusalem, Yerushalayim, Israel; 2 Hadassah Hebrew University Hospital, Mt Scopus Jerusalem, Israel, Jerusalem, Israel

## Abstract

Although the impact of visual impairment (VI) upon functional status and mortality among older people is recognized, its relationship to cognitive function is unclear. We examined the association between VI and subsequent cognitive decline from age 85-90 among subjects from the Jerusalem Longitudinal Study (1990-2020), which follows a representative study sample born 1920-21. Assessment at age 85 (2005) and age 90 (2010) included Snellen visual testing and Mini Mental State Examination (MMSE) for 488 subjects. VI was defined as corrected best eye vision ≤ 40/60. Dementia was defined as MMSE ≤24/30, after visually-dependent items (drawing, writing, reading) were excluded from the MMSE, and the score (maximum=27) was corrected to a maximum of 30 by multiplying by a factor of 1.111. At age 85 frequency of VI was 40.1% (198/488) and 86.9% (424/488) of subjects were non-demented (MMSE ≥24/30). Between ages 85-90 the mean decline in MMSE among all subjects with VI vs. non-VI was 5.2±7.7 vs. 3.9±6.7 (p=0.053), among non-demented subjects was 5.2±7.8 vs. 3.5±6.3 (p=0.002), and the transition to dementia was 34% vs. 20% (p=0.004) respectively. In logistic regression analysis among non-demented subjects at age 85, the unadjusted odds ratio (OR) for transition to dementia by age 90 associated with VI at age 85 was OR 1.95, 95%CI 1.24-3.06, p<0.01, and after adjusting for gender, years of education, depression and diabetes, the adjusted OR was 1.74, 95%CI 1.09-2.76, p<0.05. In conclusion, visual impairment at age 85 is independently associated with subsequent cognitive decline and the development of dementia.

